# Supplementary material for: Infiltrating natural killer cells bind, lyse and increase chemotherapy efficacy in glioblastoma stem-like tumorospheres
Source: Commun Biol. 2022 May 10;5:436. doi: 10.1038/s42003-022-03402-z (PMC9090761; doi:10.1038/s42003-022-03402-z)
Supplement: Supplementary file 2 — Supplementary Information [file 42003_2022_3402_MOESM2_ESM.pdf]

Supplementary file

Infiltrating natural killer cells bind, lyse and increase chemotherapy efficacy in glioblastoma stem-like tumorospheres

By authors: Barbara Breznik, Meng-Wei Ko, Christopher Tse, Po-Chun Chen, Emanuela Senjor, Bernarda Majc, Anamarija Habič, Nicolas Angelillis, Metka Novak, Vera Župunski, Jernej Mlakar, David Nathanson, Anahid Jewett

Materials

Reagents and antibodies

Human recombinant IL-2 was obtained from NIH-BRB. Monoclonal antibodies against CD16, recombinant human IFN-γ, M-CSF, conjugated primary antibodies against CD3/16/56, CD45, CD19, CD14, CD4, CD8, CD3, HLA-DR (MHC class II), CD44, CD54, MHC class I, PD-L1 (B7-H1), CD155, MICA/B, CD112, HLA-E, unconjugated primary antibodies against GFAP and PE-conjugated anti-mouse secondary antibodies were purchased from Biolegend (San Diego, CA, USA). Monoclonal antibodies against CD16, CD45, ULBP1, ULBP3 and ULBP2/5/6 and polyclonal antibodies against CD56 were purchased from R&D systems (Minneapolis, Minnesota, USA). Monoclonal antibodies against CD3/28 was obtained from Stemcell Technologies (Vancouver, Canada). Monoclonal antibodies against SOX2, CD3, OLIG2, CD8alfa and NCR1 and polyclonal antibodies against SMA, GFAP and CD133 were purchased from Abcam (Cambridge, UK). Monoclonal antibodies against CD44 was purchased from Bio-Rad (Hercules, CA, USA). Polyclonal donkey anti-goat 488, anti-rabbit 546, anti-mouse 647 antibodies, polyclonal goat anti-mouse 488 antibodies and ProLong Gold Antifade Mountant were purchased from Invitrogen (ThermoFisher Scientific, Waltham, MA, USA). Monoclonal antibodies against B7-H6 were purchased from Invitrogen (ThermoFisher Scientific). TrueBlack Lipofuscin Autofluorescence Quencher was purchased from Biotium Inc. (Hayward, CA, USA). Hoechst 33258 solution, propidium iodide (PI) and temozolomide

(TMZ) were purchased from Sigma-Aldrich (St. Louis, MO, USA). Trypsin-EDTA, TrypLE Express enzyme, Collagenase type II, CellTracker green CMFDA and CellTracker blue CMAC were obtained from Life Technologies (ThermoFisher Scientific, Waltham, MA, USA). Cisplatin (CDDP or cis-diamminedichloridoplatinum (II)) was purchased from Ronald Reagan UCLA Medical Center Pharmacy (Los Angeles, CA, USA). RANKL was purchased from PeproTech (NJ, USA).

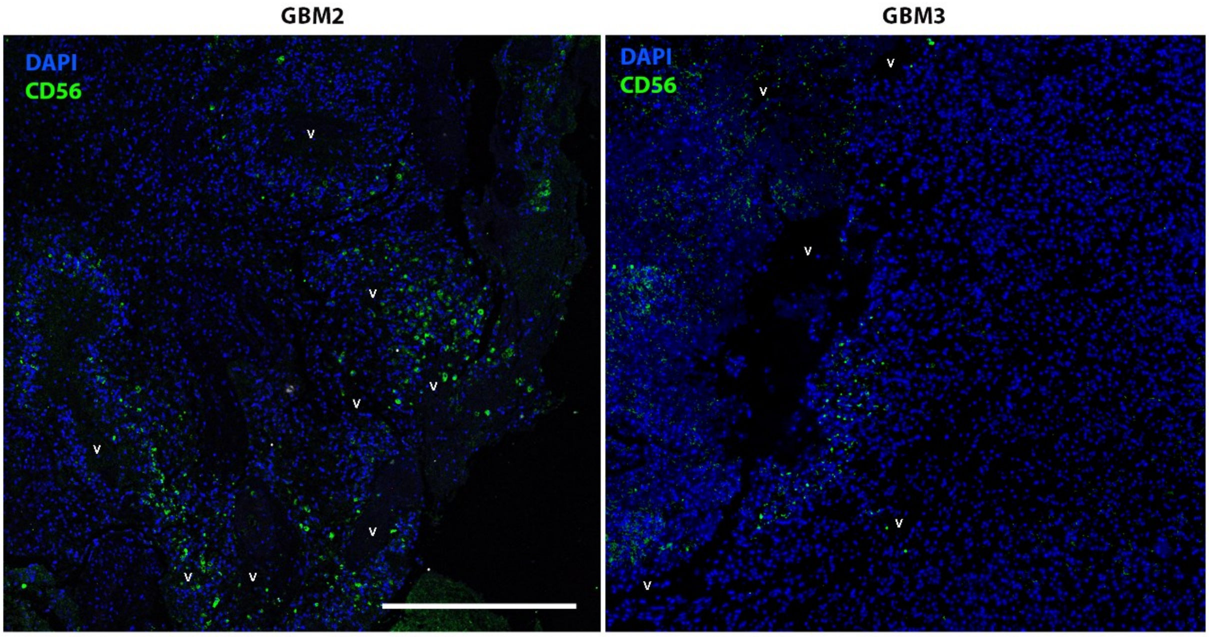

Supplementary Figure 1. Abundance of CD56 NK cell marker immunostaining in GBM tissues is low. CD56-positive cells (green) were found only in few areas in GBM tissues around vessels (v). Scale bar = 500 µm.

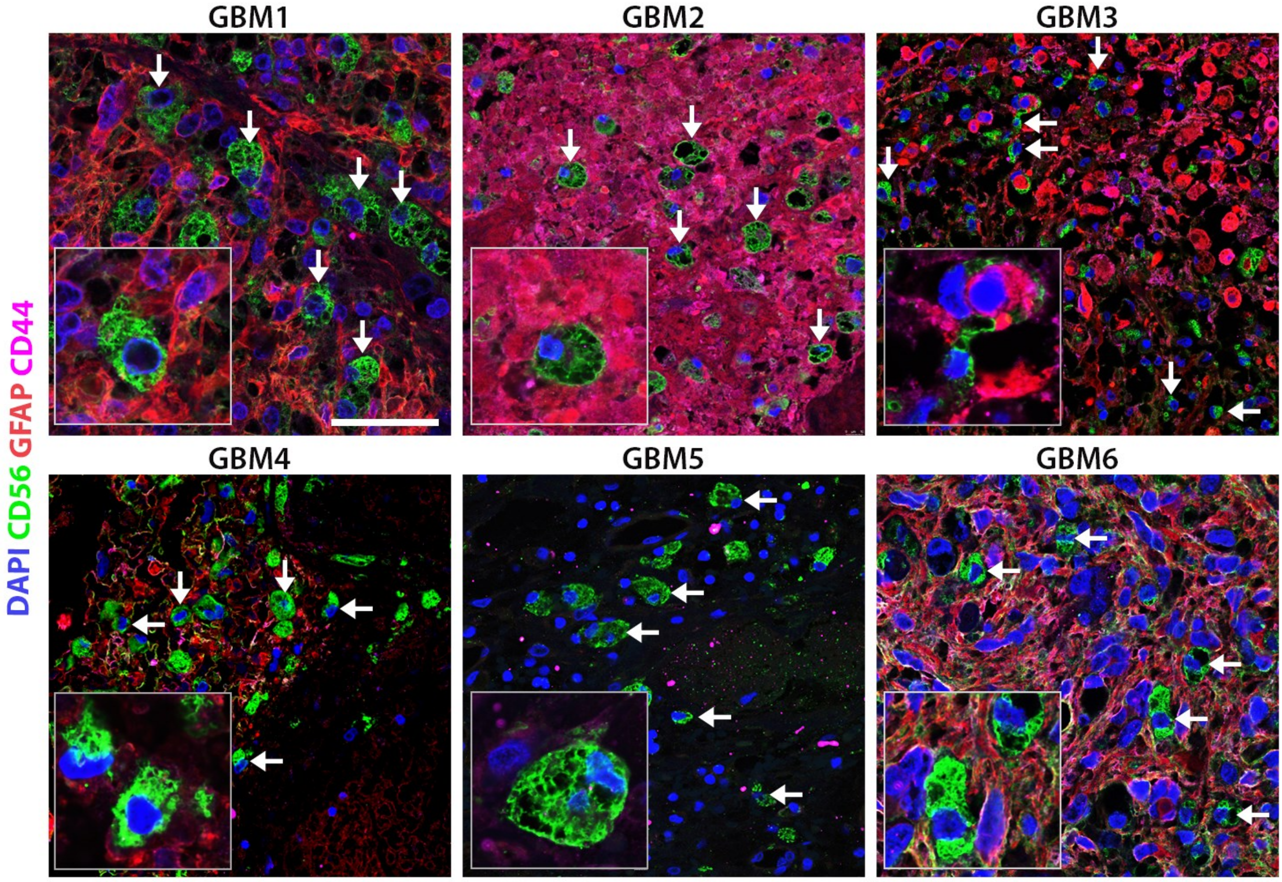

Supplementary Figure 2. CD56-positive cells did not express GFAP and CD44 in 6 GBM tissues and were in close proximity of GFAP and CD44-positive cells. NK cells were defined as CD56(green)-positive and GFAP(red)- and CD44(magenta)-negative cell population indicated by arrows. Scale bar = 50 μm.

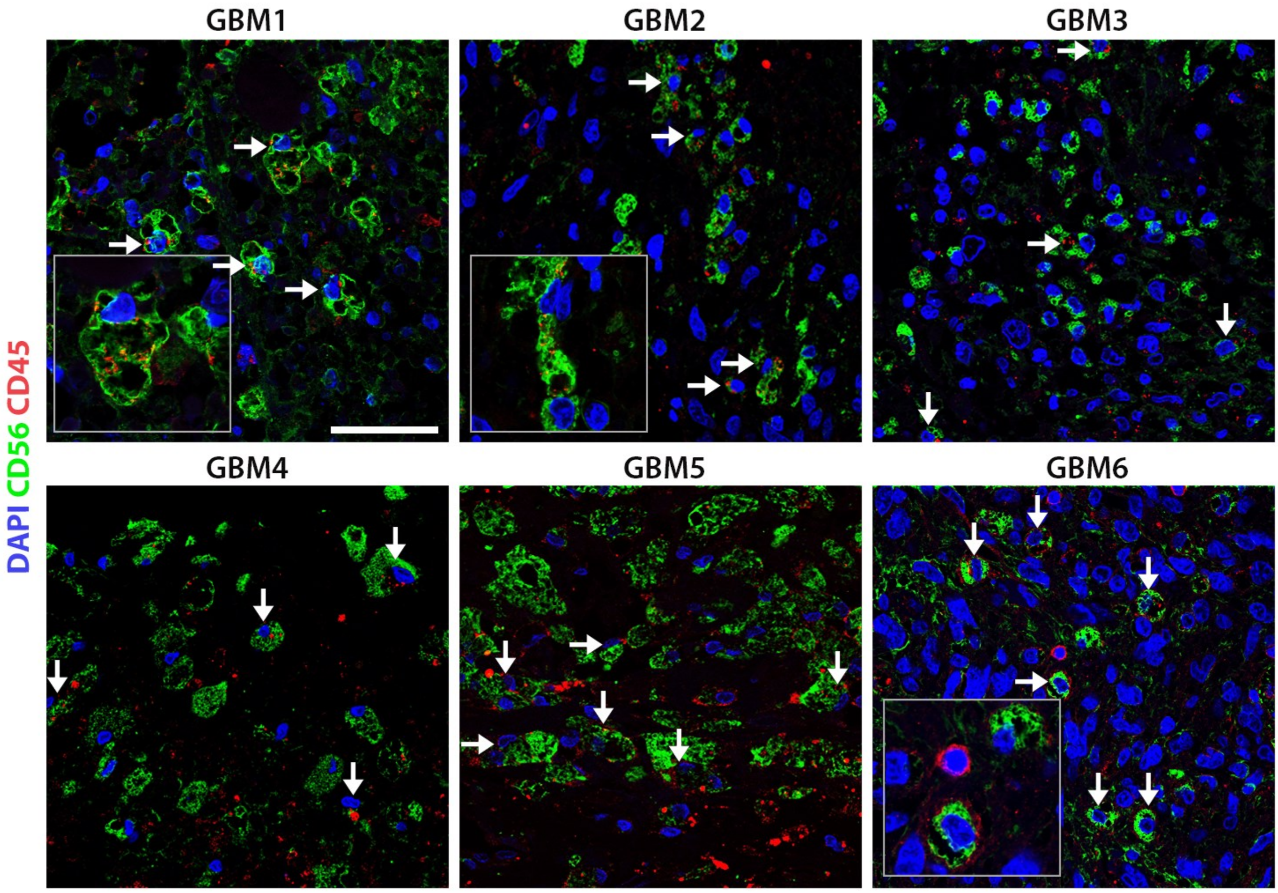

Supplementary Figure 3. CD56- and CD45-positive NK cells were detected in GBM tissues of 6 patients. NK cells were defined as CD56(green)- and CD45(red)-positive cell population indicated by arrows. Scale bar = 50 µm..

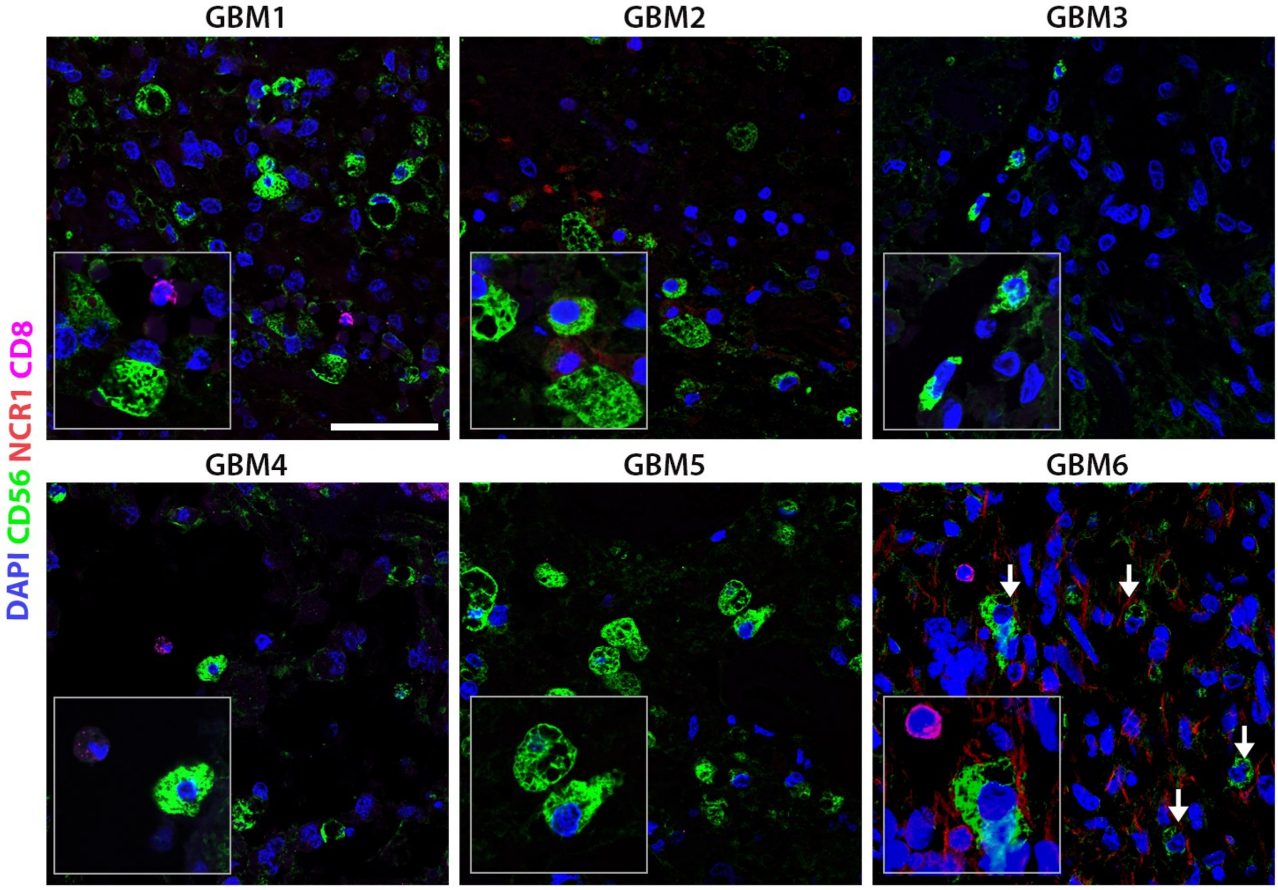

Supplementary Figure 4. CD56-positive cells were negative for CD8 marker (purple) in GBM tissues. CD56-positive cells were negative for NCR1 (NKp46, red) marker in most GBM tissues except GBM6, where CD56-positive cells expressed also NCR1 (indicated by arrows). NK cells were defined as CD56(green)-positive cell population. Scale bar = 50 μm.

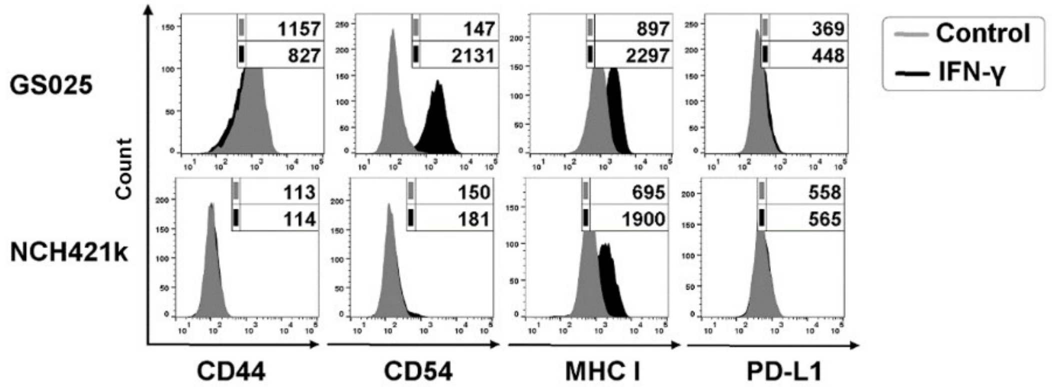

Supplementary Figure 5. IFN-γ treatment increased expression of cell surface proteins CD54 and MHC class I in GSLCs. After GSLCs were treated with 30 ng of recombinant human IFN-γ for 48h, cell surface proteins expression was analyzed by flow cytometry. Histograms show mean fluorescence intensity of control (gray) and IFN-γ-treated (black histograms) cells. The numbers in right-hand corner are the mean channel fluorescence intensities. IgG staining was used as control.

a

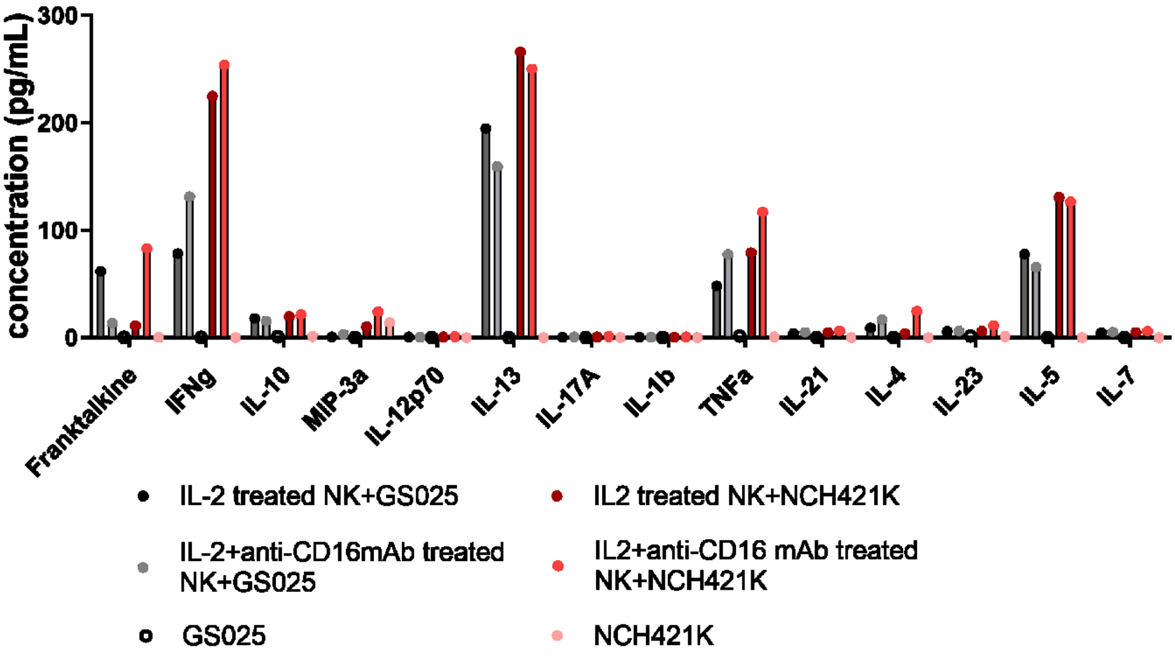

b

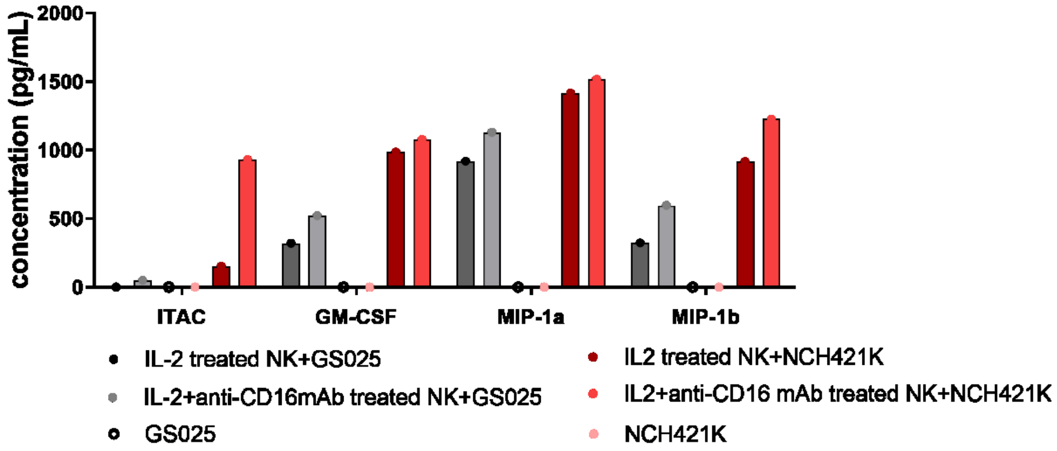

Supplementary Figure 6. Cytokine and chemokine secretion in GSLC tumorospheres after treatment with super-charged NK cells. After super-charged NK cells (IL-2 treated) or split anergized super-charged NK cells (IL-2 and anti-CD16 mAb treated) were added to tumoropsheres of GS025 (grey bars) and NCH421k (red bars) GSLCs for 24h multiplex cytokine assay was performed for 14 (**a**) and 4 (**b**) different cytokines and chemokines. Data points represent each independent biological measurement.

	CSF
**NK cells (CD56+CD16+CD3-)**	19.5 %
**CD3+4+ cells**	14.3 %
**CD3+8+ cells**	19.6 %
**CD19+ cells**	18.7 %
**CD14+ cells**	21.2 %
**total**	93.3 %

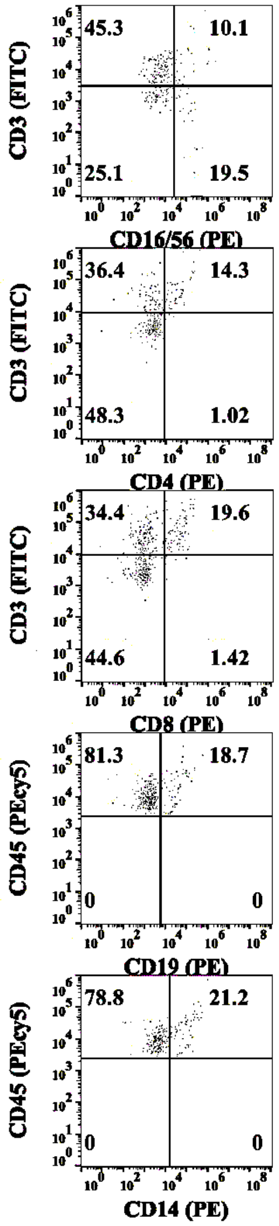

Supplementary Figure 7. Immune cell percent in cerebrospinal fluid (CSF) of patient with GBM. Immune cell percent within CD45-positive population was determined by immunolabeling and flow cytometry. The study as well as the procedures were approved by the UCLA Institutional Review Board (IRB#11-000781), and all participants signed written informed consent in accordance with the Declaration of Helsinki. Patient´s physician performed the procedure of CSF tapping by lumbar puncture according to the clinical protocol in a hospital. A total volume of 10 mL of CSF was collected in a tube without any medium and processed within 1-3 hours from collection to minimize cell loss. There were no signs of

peripheral blood contamination in CSF in the test tube, when CSF arrived in the laboratory. CSF was utilized for flow cytometry analysis. The CSF was spun at 1000 rpm for 5 min, the supernatant fluid was discarded and the cell pellet was suspended in 1x PBS and stained with antibodies against CD45, CD3/CD16/CD56, CD3, CD8, CD4, CD14 and CD19, and analyzed using flow cytometry.

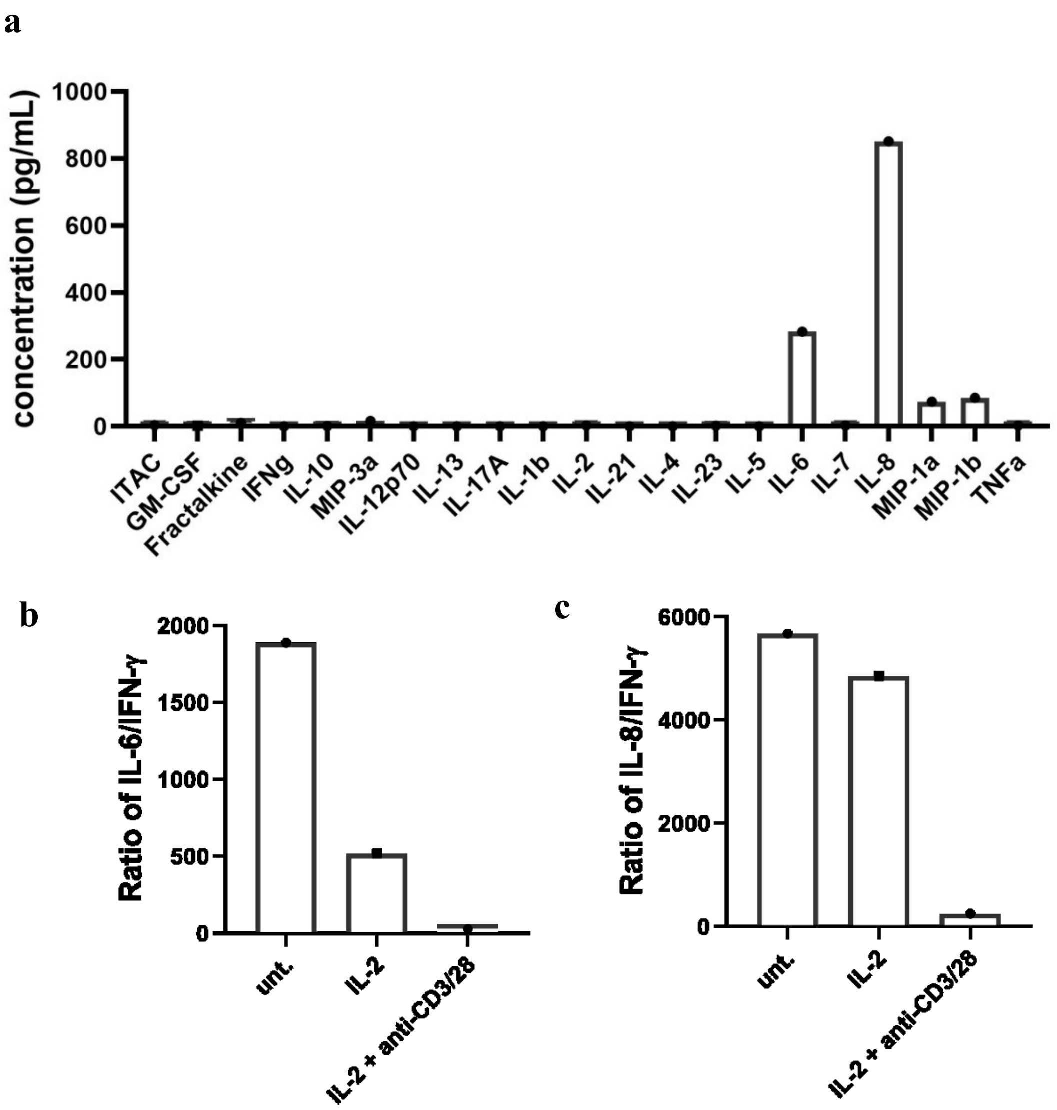

Supplementary Figure 8. Cytokine and chemokine levels in CSF of GBM patient. Concentrations of 21 cytokines and chemokines in CSF of GBM patient were measured by Multiplex cytokine assay (**a**). Ratios of IL-6 to IFN-γ (**b**) and IL-8 to IFN-γ (**c**) levels were assessed in untreated and treated CSF with IL-2 or IL-2 and anti-CD3/28 mAb. Data points represent each independent biological measurement. Data were taken from Supplementary Table 1.

Supplementary Table 1. Cytokine and chemokine levels in CSF of GBM patient. Concentrations of 21 cytokines and chemokines in CSF of GBM patient were measured by Multiplex cytokine assay. Cytokine and chemokine concentrations (pg/mL) were determined in untreated and treated CSF with IL-2 or IL-2 and anti-CD3/28 mAb.

	Concentrations (pg/mL)																				
	**GM-CSF**	**MIP-3a**	**IL-6**	**IL-8**	**MIP-1a**	**MIP-1b**	**TNFa**	**ITAC**	**Franktalkine**	**IFNg**	**IL-10**	**IL-12p70**	**IL-13**	**IL-17A**	**IL-1b**	**IL-21**	**IL-4**	**IL-23**	**IL-5**	**IL-7**
**CSF untreated**	**2.37**	**16.07**	**283.11**	**851.1**	**74.01**	**85.6**	**3.2**	**3.54**	**10.38**	**0.15**	**1.38**	**0.16**	**0.05**	**0.31**	**0.28**	**0.27**	**0.07**	**1.8**	**0.21**	**3.03**
**CSF IL-2 treated**	**185.81**	**157.95**	**202.14**	**1892**	**913.94**	**192.86**	**69.4**	**2.15**	**10.88**	**0.39**	**11.54**	**0.27**	**0.11**	**0.6**	**3.85**	**0.68**	**0.8**	**2.61**	**0.25**	**3.27**
**CSF IL-2 + anti-CD3/28 treated**	**299.25**	**186.76**	**251.58**	**2153**	**1104**	**255.92**	**91.97**	**29.11**	**19.17**	**8.75**	**14.77**	**0.38**	**0.15**	**0.6**	**0.8**	**1.65**	**2.27**	**3.96**	**0.26**	**3.78**

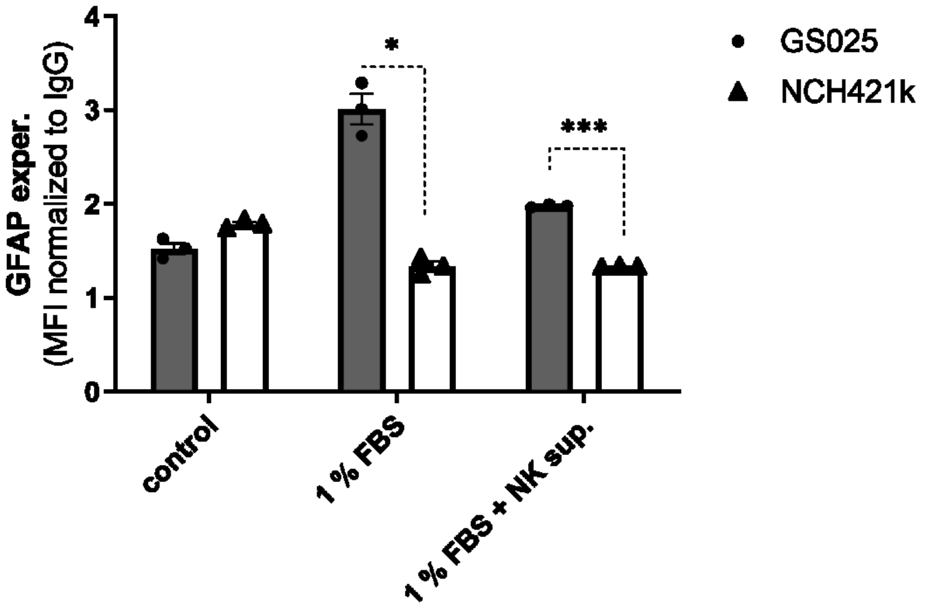

Supplementary Figure 9. Increased GFAP expression in GS025 GSLCs after exposure to 1 % FBS and NK supernatant. 1 % FBS or 1% FBS and NK supernatant increased GFAP expression in GS025 GSLCs (grey bars) in comparison to NCH421k GSLCs (white bars). Mean fluorescence intensities normalized to IgG control were measured after 6 days of exposure by flow cytometry. Data are represented as means ± SEM (n=3, independent biological replicates). * $p < 0.05$, *** $p < 0.001$.

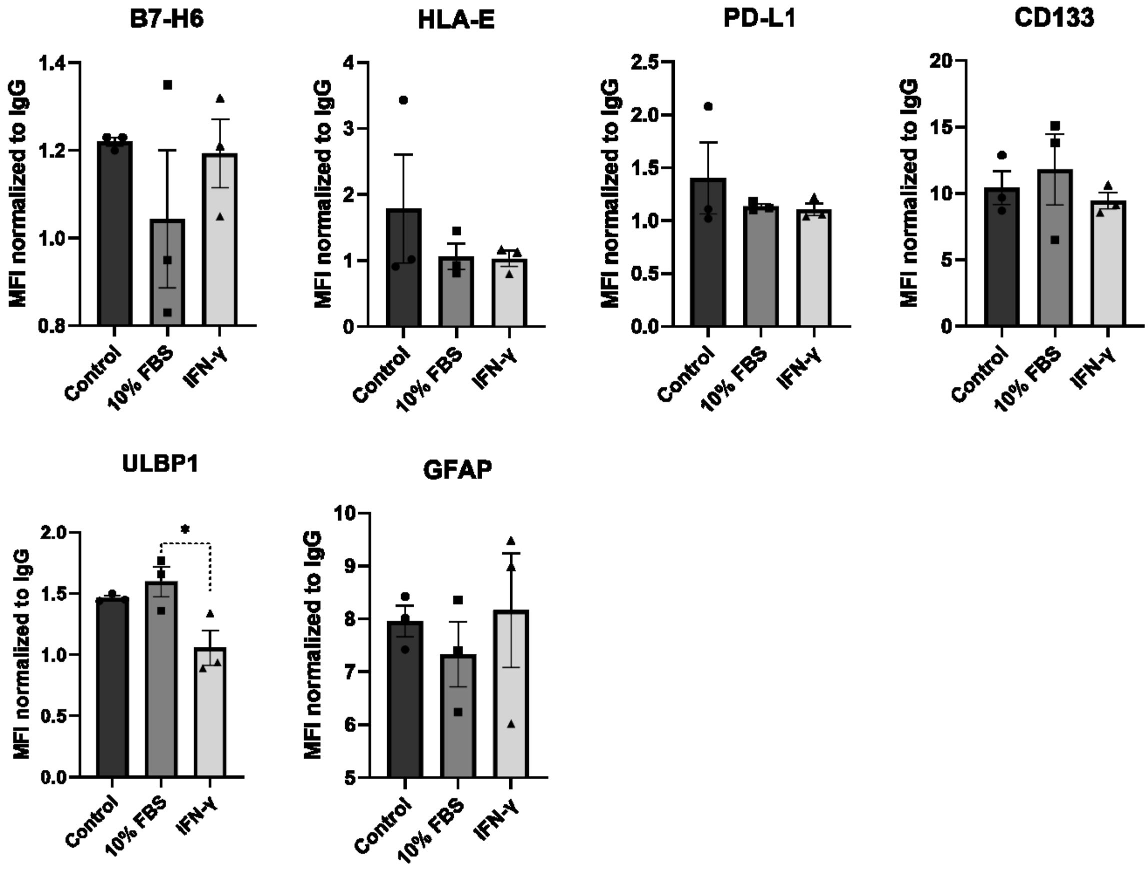

Supplementary Figure 10. Protein expression of B7-H6, HLA-E, PD-L1. CD133, ULBP1 and GFAP in NCH421k cells after treatment with culture media supplemented with 10% FBS or IFN-γ. After NCH421k cells were treated with cell culture medium containing 10 % FBS (dark grey bars) for 7 days or 30 ng of recombinant human IFN-γ (light grey bars) for 48h, protein expression was analyzed by flow cytometry. Culture medium was used as control (black bars). Mean fluorescence intensity (MFI) was measured and normalized to antibody controls. Data are presented as means ± SEM (n=3, independent biological replicates).

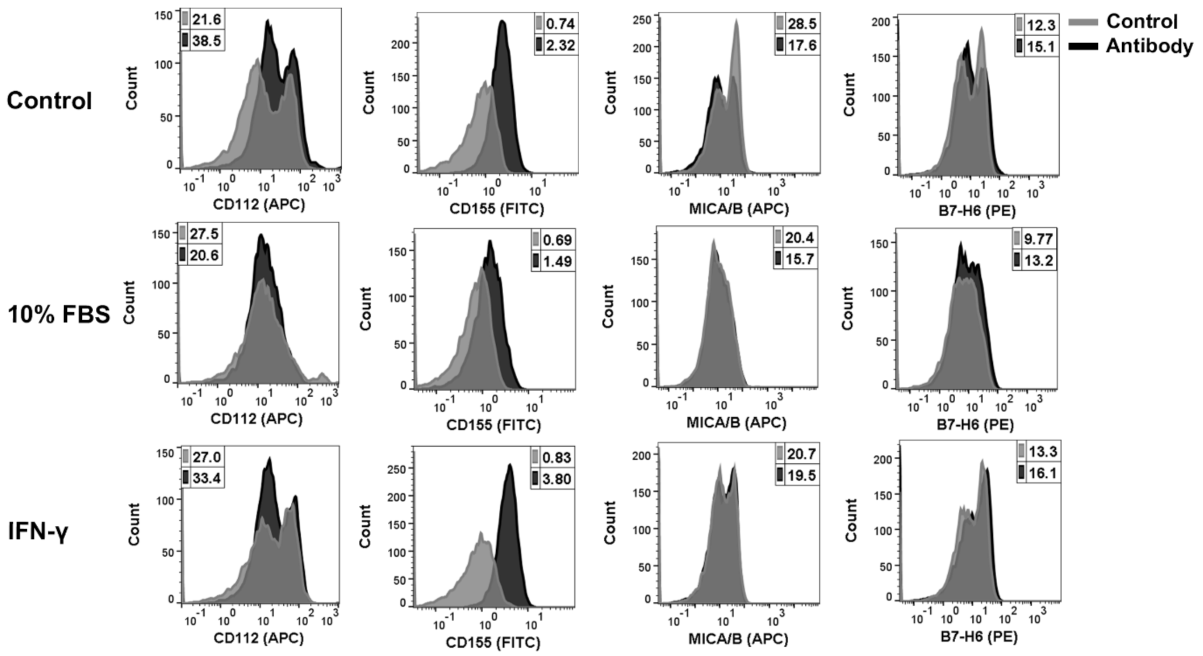

Supplementary Figure 11a. Cell surface expression of ligands for activating NK cell receptors CD112, CD155, MICA/B and B7-H6 in GSLCs after treatment with 10 % FBS and IFN-γ. After NCH421k cells were treated with cell culture medium containing 10 % FBS for 7 days or 30 ng of recombinant human IFN-γ for 48h, cell surface proteins expression was analyzed by flow cytometry. Histograms show mean fluorescence intensity of controls (gray) and antibody stained cells (black). The numbers in right(left)-hand corner are the mean channel fluorescence intensities. IgG staining was used as control.

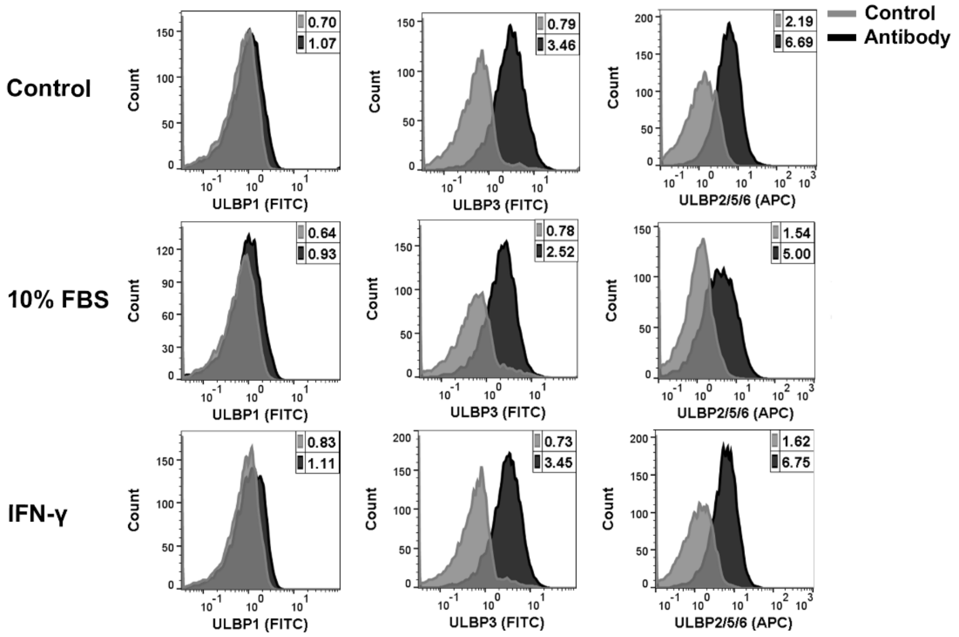

Supplementary Figure 11b. Cell surface expression of ligands for activating NK cell receptors ULBP1, ULBP3 and ULBP2/5/6 in GSLCs after treatment with 10 % FBS and IFN-γ. Continuation of supplementary Figure 11a.

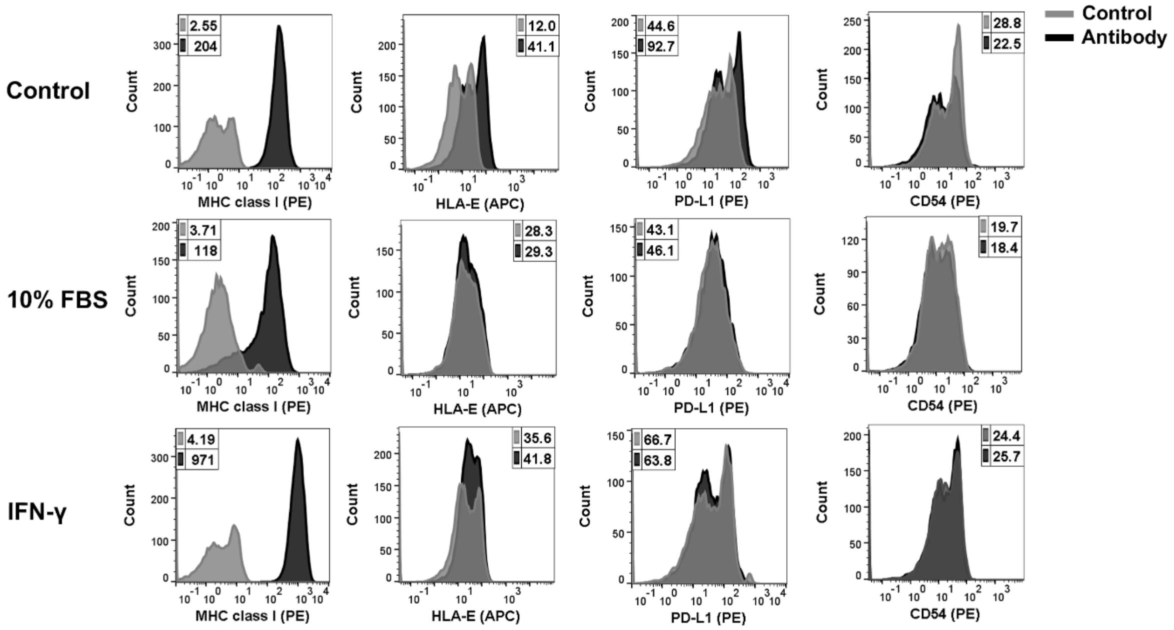

Supplementary Figure 12. Cell surface expression of ligands for inhibitory NK cell receptors MHC class I, HLA-E, PD-L1, CD54 in GSLCs after treatment with 10 % FBS and IFN-γ. After NCH421k cells were treated with cell culture medium containing 10 % FBS for 7 days or 30 ng of recombinant human IFN-γ for 48h, cell surface proteins expression was analyzed by flow cytometry. Histograms show mean fluorescence intensity of controls (gray) and antibody stained cells (black). The numbers in right(left)-hand corner are the mean channel fluorescence intensities. IgG staining was used as control.

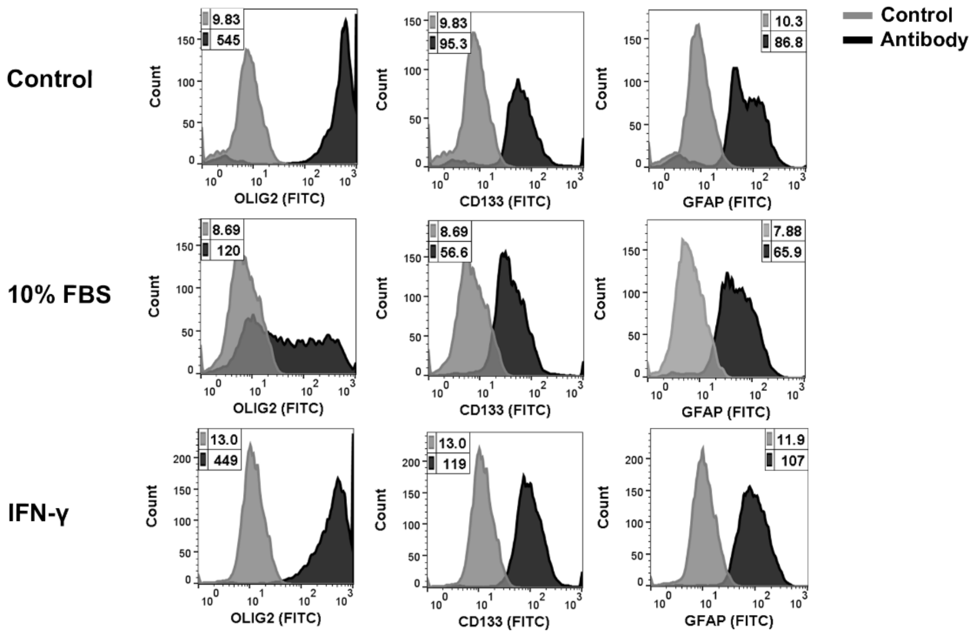

Supplementary Figure 13. Expression of GSLC markers OLIG2 and CD133 and astrocytic marker GFAP after treatment with 10 % FBS and IFN-γ. After NCH421k cells were treated with cell culture medium containing 10 % FBS for 7 days or 30 ng of recombinant human IFN-γ for 48h, intracellular staining was performed and protein expression was analyzed by flow cytometry. Histograms show mean fluorescence intensity of controls (gray) and antibody stained cells (black). The numbers in right(left)-hand corner are the mean channel fluorescence intensities. Staining in the absence of primary antibodies was used as control.

Supplementary Table 2. Details of the 8 GBM patients and their tumors, operated at the Department of Neurosurgery of the University Medical Centre Ljubljana, Slovenia.

**Patient**	**Sex**	**Age (years)**	**Overall survival (months) ***	**IDH1 R132H mutation (yes/no)****	**p53 mutation (yes/no)*****	**ATRX*******	**Tumor site/side*********	**NK cells (yes/no) ***********	**Therapy (radio- or chemotrerapy with TMZ)**
**GBM1**	**M**	**66**	**8**	**no**	**n**	**n.d.**	**Temporal / l**	**yes**	Radiotherapy (60 Gy) + TMZ, adjuvant TMZ
**GBM2**	**M**	**54**	**3**	**no**	**n**	**n.d.**	**Temporal-occ / r**	**yes**	Radiotherapy (60 Gy) + TMZ, adjuvant TMZ
**GBM3**	**M**	**43**	**20**	**yes**	**yes**	**n.d.**	**Frontal / l**	**yes**	Radiotherapy (60 Gy) + TMZ, adjuvant TMZ
**GBM4**	**M**	**62**	**17**	**no**	**no**	**loss**	**Temporal / r**	**yes**	Radiotherapy (60 Gy) + TMZ, adjuvant TMZ
**GBM5**	**M**	**46**	**16**	**no**	**yes**	**n.d.**	**Temporal / r**	**yes**	Radiotherapy (60 Gy) + TMZ, adjuvant TMZ
**GBM6**	**M**	**55**	**26**	**no**	**no**	**n.d.**	**Temporal / r**	**yes**	Radiotherapy (60 Gy) + TMZ, adjuvant TMZ
**GBM7**	**F**	**59**	**16**	**no**	**n**	**n.d.**	**Frontal / r**	**no**	Radiotherapy (60 Gy) + TMZ, adjuvant TMZ
**GBM8**	**M**	**71**	**12**	**no**	**no**	**wt**	**Temporal / r**	**no**	Radiotherapy (30 Gy), adjuvant TMZ

**Overall survival was determined as the period in months from the date of the first operation until death of the patient*
***IDH: Isocitrate dehydrogenase enzyme mutations were determined at the Pathology,*
****p53 status was determined at the Pathology; yes/no/n=not known*
*****ATRX mutation was determined at the Pathology; n.d.=not determined, loss, wt=wild type*
******Site/Side: Frontal/Temporal/occ-Occipital lobe; r=right, l=left*
*******NK cells present: yes/no*

Supplementary Table 3. Details of antibodies used for fluorescence immunohistochemistry.

Antibodies	Catalog/Lot Number, Company	Concentration/dilution
**Primary antibodies**		
**Goat anti-human CD56 antibody**	Cat #AF2408, Lot VOK0219021, R&D systems	10 μg/mL
**Mouse anti-human CD16 antibody,** clone 1001049	Cat #MAB4325, Lot CLYI0119081, R&D systems	20 μg/mL
**Mouse anti-human SOX2 antibody**, clone 20G5	Cat #ab171380, Lot GR3253929-1, Abcam	1:50
**Rabbit anti-human CD3 antibody**, clone SP7	Cat #ab16669, Lot GR3285725-13, Abcam	1:150
**Rabbit anti-human alpha smooth muscle actin (SMA) antibody**	Cat # ab5694, Lot GR248336-10, Abcam	1:100
**Rabbit anti-human GFAP antibody**	Cat # ab211271, Lot GR285910-7, Abcam	1:1000
**Mouse anti-human CD44 antibody**, clone Bu52	Cat #MCA2504, Lot 1605, Bio-Rad	1:100
**Mouse anti-human CD45 antibody**, clone 2D1	Cat #MAB1430, Lot ILP0920101, R&D systems	10 μg/mL
**Mouse anti-human CD8 alfa antibody,** C8/144B	Cat# ab17147, Lot GR3395232-3, Abcam	1:100
**Rabbit anti-human NCR1 (NKp46) antibody,** EPR22403-57	Cat# ab224703, Lot GR3400107-2, Abcam	1:500
**Secondary antibodies**		
**Alexa Fluor 488-conjugated donkey anti-goat antibody**	Cat #A32814, Lot # UI289709, ThermoFisher Scientific	1:200
**Alexa Fluor 546-conjugated donkey anti-rabbit antibody**	Cat #A10040, Lot #948483, ThermoFisher Scientific	1:200
**Alexa Fluor 647-conjugated donkey anti-mouse antibody**	Cat #A32787, Lot #UI291059 ThermoFisher Scientific	1:200

Supplementary Table 4. Details of antibodies used for flow cytometry.

Antibodies	Catalog/Lot Number, Company	Concentration/dilution
**Primary antibodies**		
**PE/Cyanine5-conjugated mouse anti-human CD45 Antibody**, clone HI30	Cat #304010, Lot B284913, Biolegend	1:20
**FITC-conjugated mouse anti-human CD3 Antibody**, clone UCHT1	Cat #300440, Lot B279209, Biolegend	1:20
**PE-conjugated mouse anti-human CD8a Antibody**, clone HIT8a	Cat #300908, Lot B250825, Biolegend	1:20
**Mouse anti-human CD3 FITC/(CD16+CD56) PE Cocktail**, clone UCHT1; 3G8; MEM-188;	Cat #319101, Lot B269557, Biolegend	1:20
**PE-conjugated mouse anti-human CD14 Antibody**, clone 63D3	Cat #367104, Lot B274117, Biolegend	1:20
**PE-conjugated mouse anti-human CD19 Antibody**, clone HIB19	Cat # 302208, Lot B273506, Biolegend	1:20
**PE-conjugated mouse anti-human CD44 Antibody**, clone BJ18	Cat #338808, Lot B297282, Biolegend	1:20
**PE-conjugated mouse anti-human CD4 Antibody,** clone RPA-T4	Cat #300508, Biolegend	1:20
**PE-conjugated mouse anti-human CD54 Antibody**, clone HA58	Cat #353106, Lot B243651, Biolegend	1:20
**PE-conjugated mouse anti-human PD-L1 (CD274, B7-H1) Antibody,** clone 29E.2A3	Cat #329706, Lot B272166, Biolegend	1:20
**PE-conjugated mouse anti-human MHC I (HLA-A,B,C) Antibody**, clone W6/32	Cat #311406, Lot B265864, Biolegend	1:20
**Purified mouse anti-human anti-GFAP Antibody**, clone 2E1.E9	Cat #644701, Lot B27479, Biolegend	1:500
**Recombinant rabbit anti-human Anti-Olig2 antibody**, clone EPR2673	Cat #ab109186, Lot GR3359224-6, Abcam	1:200
**Rabbit anti-human Anti-CD133 antibody - Stem Cell Marker**	Cat # ab19898, Lot GR3215691-1, Abcam	1:200
**FITC mouse anti-human CD155 (PVR) Antibody**	Cat # 337627, Lot B317987, Biolegend	1:20
**B7-H6 mouse anti-human Monoclonal Antibody (JAM1EW), PE, eBioscience™ Antibody**	Cat # 12-6526-41, Lot 2354930, eBioscience, ThermoFisherScientific	1:20
**APC mouse anti-human MICA/MICB Antibody**	Cat # 320907, Lot B312970, Biolegend	1:20
**Mouse anti-human ULBP-1 Alexa Fluor® 488-conjugated Antibody**	Cat # FAB1380G, Lot ADBF0321091, R&D Systems	1:20
**Mouse anti-human ULBP-2/5/6 APC-conjugated Antibody**	Cat # FAB1298A, Lot LWF0620021, R&D Systems	1:10
**Mouse anti-human ULBP-3 Antibody**	Cat # MAB1517-SP, Lot JFY022110A, R&D Systems	1:20
**APC mouse anti-human CD112 (Nectin-2) Antibody**	Cat # 337411, Lot B284882, Biolegend	1:20

**APC mouse anti-human HLA-E Antibody**	Cat # 342605, Lot B304555, Biolegend	1:20
**Secondary antibodies**		
**PE-conjugated goat anti-mouse IgG (minimal x-reactivity) Antibody**, clone Poly4053	Cat #405307, Lot B253778, Biolegend	1:200
**Goat anti-Rabbit IgG (H+L) Cross-Adsorbed Secondary Antibody, Alexa Fluor 488**	Cat # A1108, ThermoFisher Scientific	1:200
