# Supplementary material for: Infiltrating natural killer cells bind, lyse and increase chemotherapy efficacy in glioblastoma stem-like tumorospheres
Source: Commun Biol. 2022 May 10;5:436. doi: 10.1038/s42003-022-03402-z (PMC9090761; doi:10.1038/s42003-022-03402-z)
Supplement: Supplementary file 3 — Description of Additional Supplementary Files [file 42003_2022_3402_MOESM3_ESM.pdf]

Description of Additional Supplementary Files

File name: Supplementary Data 1

Description: Source data for the graphs and charts presented in the figures in main text and supplements.
