# Supplementary material for: Infiltrating natural killer cells bind, lyse and increase chemotherapy efficacy in glioblastoma stem-like tumorospheres
Source: Commun Biol. 2022 May 10;5:436. doi: 10.1038/s42003-022-03402-z (PMC9090761; doi:10.1038/s42003-022-03402-z)
Supplement: Supplementary file 5 — Reporting Summary [file 42003_2022_3402_MOESM5_ESM.pdf]

nature research

Corresponding author(s): Barbara Breznik, Anahid Jewett

Last updated by author(s): April 6, 2022

Reporting Summary

Nature Research wishes to improve the reproducibility of the work that we publish. This form provides structure for consistency and transparency in reporting. For further information on Nature Research policies, see our Editorial Policies and the Editorial Policy Checklist.

Statistics

For all statistical analyses, confirm that the following items are present in the figure legend, table legend, main text, or Methods section.

n/a	Confirmed	
☐	☒	The exact sample size (n) for each experimental group/condition, given as a discrete number and unit of measurement
☐	☒	A statement on whether measurements were taken from distinct samples or whether the same sample was measured repeatedly
☐	☒	The statistical test(s) used AND whether they are one- or two-sided<br>*Only common tests should be described solely by name; describe more complex techniques in the Methods section.*
☒	☐	A description of all covariates tested
☒	☐	A description of any assumptions or corrections, such as tests of normality and adjustment for multiple comparisons
☐	☒	A full description of the statistical parameters including central tendency (e.g. means) or other basic estimates (e.g. regression coefficient) AND variation (e.g. standard deviation) or associated estimates of uncertainty (e.g. confidence intervals)
☒	☐	For null hypothesis testing, the test statistic (e.g. F, t, r) with confidence intervals, effect sizes, degrees of freedom and P value noted<br>*Give P values as exact values whenever suitable.*
☒	☐	For Bayesian analysis, information on the choice of priors and Markov chain Monte Carlo settings
☒	☐	For hierarchical and complex designs, identification of the appropriate level for tests and full reporting of outcomes
☒	☐	Estimates of effect sizes (e.g. Cohen's d, Pearson's r), indicating how they were calculated

Our web collection on statistics for biologists contains articles on many of the points above.

Software and code

Policy information about availability of computer code

Data collection	LAS X Life Sciences software (Leica), ImmunoSpot® SOFTWARE (CTL Europe GmbH), ZEN blue software (Zeiss), Imaris (Bitplane) software version 9.5.1 (Oxford Instruments), Attune NxT software (Thermo Fisher Scientific)
Data analysis	GraphPad Prism 8.0 was used to analyze data for this study.

For manuscripts utilizing custom algorithms or software that are central to the research but not yet described in published literature, software must be made available to editors and reviewers. We strongly encourage code deposition in a community repository (e.g. GitHub). See the Nature Research guidelines for submitting code & software for further information.

Data

Policy information about availability of data

All manuscripts must include a data availability statement. This statement should provide the following information, where applicable:

- Accession codes, unique identifiers, or web links for publicly available datasets
- A list of figures that have associated raw data
- A description of any restrictions on data availability

All data generated or analyzed during this study are included in this published article and its supplementary information files. The datasets generated during and/or analyzed during the current study are also available from the corresponding author on reasonable request.

Field-specific reporting

Please select the one below that is the best fit for your research. If you are not sure, read the appropriate sections before making your selection.

☒ Life sciences ☐ Behavioural & social sciences ☐ Ecological, evolutionary & environmental sciences

For a reference copy of the document with all sections, see nature.com/documents/nr-reporting-summary-flat.pdf

Life sciences study design

All studies must disclose on these points even when the disclosure is negative.

Sample size	No formal statistical method was used to pre-determine sample size. Sample sizes were estimated based off pilot experiments and from previous literature. All experiments were set up in duplicates or triplicates and tested in at least 3 independent experiment unless otherwise stated. Independent means that experiments were repeated with 3 different passages of cell cultures and 3 different patients/animals for evaluation of immune cell function and in vivo tumor growth, respectively.
Data exclusions	No data were excluded from analyses.
Replication	All attempts at replication were successful.
Randomization	Samples were allocated into experimental groups based on simple randomization. This technique maintains complete randomness of the assignment of a subject to a particular group. Groups with similar number of samples were generated.
Blinding	Investigators were not blinded to group allocation.

Reporting for specific materials, systems and methods

We require information from authors about some types of materials, experimental systems and methods used in many studies. Here, indicate whether each material, system or method listed is relevant to your study. If you are not sure if a list item applies to your research, read the appropriate section before selecting a response.

Materials & experimental systems

n/a	Involved in the study	
☐	☒	Antibodies
☐	☒	Eukaryotic cell lines
☒	☐	Palaeontology and archaeology
☐	☒	Animals and other organisms
☐	☒	Human research participants
☒	☐	Clinical data
☒	☐	Dual use research of concern

Methods

n/a	Involved in the study	
☒	☐	ChIP-seq
☐	☒	Flow cytometry
☒	☐	MRI-based neuroimaging

Antibodies

Antibodies used

Flow cytometry
-PE/Cyanine5-conjugated mouse anti-human CD45 Antibody (Cat #304010, Lot B284913, dilution 1:20, Biolegend), clone HI30
Validation statement of manufacturer: https://www.biolegend.com/en-us/products/pe-cyanine5-anti-human-cd45-antibody-709?GroupID=BLG5926
-FITC-conjugated mouse anti-human CD3 Antibody (Cat #300440, Lot B279209, dilution 1:20, Biolegend), clone UCHT1
Validation statement of manufacturer: https://www.biolegend.com/en-us/products/fitc-anti-human-cd3-antibody-863
-PE-conjugated mouse anti-human CD8a Antibody (Cat #300908, Lot B250825, dilution 1:20, Biolegend), clone HIT8a
-mouse anti-human CD3 FITC/(CD16+CD56) PE Cocktail (Cat #319101, Lot B269557, dilution 1:20, Biolegend), clone UCHT1; 3G8; MEM-188;
Validation statement of manufacturer: https://www.biolegend.com/en-us/products/anti-human-cd3-fitc-cd16-cd56-pe-cocktail-2503?GroupID=BLG13117
-PE-conjugated mouse anti-human CD14 Antibody (Cat #367104, Lot B274117, dilution 1:20, Biolegend), clone 63D3
Validation statement of manufacturer: https://www.biolegend.com/en-us/products/pe-anti-human-cd14-antibody-12011
-PE-conjugated mouse anti-human CD19 Antibody (Cat # 302208, Lot B273506, dilution 1:20, Biolegend), clone HIB19
Validation statement of manufacturer: https://www.biolegend.com/en-us/products/pe-anti-human-cd19-antibody-719
-PE-conjugated mouse anti-human CD44 Antibody (Cat #338808, Lot B297282, dilution 1:20, Biolegend), clone BJ18
Validation statement of manufacturer: https://www.biolegend.com/en-us/products/pe-anti-human-cd44-antibody-5745
-PE-conjugated mouse anti-human CD4 Antibody (Cat #300508, dilution 1:20, Biolegend), clone RPA-T4
Validation statement of manufacturer: https://www.biolegend.com/en-us/search-results/pe-anti-human-cd4-antibody-827
-PE-conjugated mouse anti-human CD54 Antibody (Cat #353106, Lot B243651, dilution 1:20, Biolegend), clone HA58
Validation statement of manufacturer: https://www.biolegend.com/en-us/products/pe-anti-human-cd54-antibody-7447

-PE-conjugated mouse anti-human PD-L1 (CD274, B7-H1) Antibody (Cat #329706, Lot B272166, dilution 1:20, Biolegend), clone 29E.2A3
Validation statement of manufacturer: https://www.biolegend.com/en-us/products/pe-anti-human-cd274-b7-h1-pd-l1-antibody-4375
-PE-conjugated mouse anti-human MHC I (HLA-A,B,C) Antibody (Cat #311406, Lot B265864, dilution 1:20, Biolegend), clone W6/32
Validation statement of manufacturer: https://www.biolegend.com/en-us/products/pe-anti-human-hla-a-b-c-antibody-1872
-Purified mouse anti-human anti-GFAP Antibody (Cat #644701, Lot B27479, dilution 1:500, Biolegend), clone 2E1.E9
Validation statement of manufacturer: https://www.biolegend.com/en-us/products/purified-anti-gfap-antibody-5753
-Recombinant rabbit anti-human Anti-Olig2 antibody (Cat #ab109186, Lot GR3359224-6, dilution 1:200, Abcam), clone EPR2673
Validation statement of manufacturer: https://www.abcam.com/olig2-antibody-epr2673-ab109186.html
- Rabbit anti-human Anti-CD133 antibody - Stem Cell Marker (Cat # ab19898, Lot GR3215691-1, dilution 1:200, Abcam)
Validation statement of manufacturer: https://www.abcam.com/cd133-antibody-stem-cell-marker-ab19898.html
- FITC mouse anti-human CD155 (PVR) Antibody (Cat # 337627, Lot B317987, dilution 1:20, Biolegend)
Validation statement of manufacturer: https://www.biolegend.com/en-us/search-results/fitc-anti-human-cd155-pvr-antibody-17877
-B7-H6 mouse anti-human Monoclonal Antibody (JAM1EW), PE, eBioscience™ Antibody (Cat # 12-6526-41, Lot 2354930, dilution 1:20, eBioscience, ThermoFisherScientific)
Validation statement of manufacturer: https://www.thermofisher.com/antibody/product/B7-H6-Antibody-clone-JAM1EW-Monoclonal/12-6526-42
-APC mouse anti-human MICA/MICB Antibody (Cat # 320907, Lot B312970, dilution 1:20, Biolegend)
Validation statement of manufacturer: https://www.biolegend.com/en-us/search-results/apc-anti-human-mica-micb-antibody-3065
-mouse anti-human ULBP-1 Alexa Fluor® 488-conjugated Antibody (Cat # FAB1380G, Lot ADBF0321091, dilution 1:20, R&D Systems)
Validation statement of manufacturer: https://www.rndsystems.com/products/human-ulbp-1-alexa-fluor-488-conjugated-antibody-170818_fab1380g
-mouse anti-human ULBP-2/5/6 APC-conjugated Antibody (Cat # FAB1298A, Lot LWF0620021, dilution 1:10, R&D Systems)
Validation statement of manufacturer: https://www.rndsystems.com/products/human-ulbp-2-5-6-apc-conjugated-antibody-165903_fab1298a
-mouse anti-human ULBP-3 Antibodydy (Cat # MAB1517-SP, Lot JFY022110A, dilution 1:20, R&D Systems)
Validation statement of manufacturer: https://www.rndsystems.com/products/human-ulbp-3-antibody-166510_mab1517
- APC mouse anti-human CD112 (Nectin-2) Antibody (Cat # 337411, Lot B284882, dilution 1:20, Biolegend)
Validation statement of manufacturer: https://www.biolegend.com/en-us/search-results/apc-anti-human-cd112-nectin-2-antibody-11898
- APC mouse anti-human HLA-E Antibody (Cat # 342605, Lot B304555, dilution 1:20, Biolegend)
Validation statement of manufacturer: https://www.biolegend.com/en-us/search-results/apc-anti-human-hla-e-antibody-10760
Secondary antibody for anti-GFAP staining:
-PE-conjugated goat anti-mouse IgG (minimal x-reactivity) Antibody (Cat #405307, Lot B253778, 1:200 dilution, Biolegend), clone Poly4053
Validation statement of manufacturer: https://www.biolegend.com/en-us/products/pe-goat-anti-mouse-igg-minimal-x-reactivity-1418
Secondary antibody for anti-OLIG2, CD133 staining:
-Goat anti-Rabbit IgG (H+L) Cross-Adsorbed Secondary Antibody, Alexa Fluor 488 (Cat # A1108, 1:200, ThermoFisher Scientific)
Validation statement of manufacturer: https://www.thermofisher.com/antibody/product/Goat-anti-Rabbit-IgG-H-L-Cross-Adsorbed-Secondary-Antibody-Polyclonal/A-11008

Isotypic controls for flow cytometry:
-Mouse IgG1/IgG1/IgG2a
-Mouse IgG1, κ
-Mouse IgG2a, κ
-Mouse IgG2b
-Mouse IgG2b, κ

Immunofluorescence
-goat anti-human CD56 antibody (Cat #AF2408, Lot #VOK0219021, concentration 10 g/mL, R&D systems)
Validation statement of manufacturer: https://www.rndsystems.com/products/human-mouse-ncam-1-cd56-antibody_af2408
-mouse anti-human CD16 antibody (Cat #MAB4325, Lot #CLYI0119081, concentration 20 g/mL, R&D systems), clone 1001049
Validation statement of manufacturer: https://www.rndsystems.com/products/human-fc-gamma-riii-cd16-antibody-1001049_mab4325
-mouse anti-human SOX2 antibody (Cat #ab171380, Lot # GR3253929-1, dilution 1:50, Abcam), clone 20G5
Validation statement of manufacturer: https://www.abcam.com/sox2-antibody-20g5-ab171380.html
-rabbit anti-human CD3 antibody (Cat #ab16669, Lot #GR3285725-13, dilution 1:150, Abcam), clone SP7
Validation statement of manufacturer: https://www.abcam.com/cd3-antibody-sp7-ab16669.html
-rabbit anti-human alpha smooth muscle actin (SMA) antibody (Cat # ab5694, Lot #GR248336-10, dilution 1:100, Abcam)
Validation statement of manufacturer: https://www.abcam.com/alpha-smooth-muscle-actin-antibody-ab5694.html
-rabbit anti-human GFAP antibody (Cat # ab211271, Lot #GR285910-7, dilution 1:1000, Abcam)
Validation statement of manufacturer: https://www.abcam.com/gfap-antibody-affinity-purified-ab211271.html
-mouse anti-human CD44 antibody (Cat #MCA2504, Lot #1605, dilution 1:100, Bio-Rad), clone Bu52
Validation statement of manufacturer: https://www.bio-rad-antibodies.com/monoclonal/human-cd44-antibody-bu52-mca2504.html?f=purified
-mouse anti-human CD45 antibody (Cat #MAB1430, Lot #ILP0920101, concentration 10 g/mL, R&D systems), clone 2D1
Validation statement of manufacturer: https://www.rndsystems.com/products/human-cd45-antibody-2d1_mab1430
-mouse anti-human CD8 alfa antibody (C8/144B, Cat# ab17147, Lot #GR3395232-3, dilution 1:100, Abcam)
Validation statement of manufacturer: https://www.abcam.com/cd8-alpha-antibody-c8144b-ab17147.html
-rabbit anti-human NCR1 (NKp46) antibody (EPR22403-57; Cat# ab224703, Lot# GR3400107-2, dilution 1:500, Abcam)
Validation statement of manufacturer: https://www.abcam.com/ncr1-antibody-epr22403-57-ab224703.html

Secondary antibodies
-Alexa Fluor 488-conjugated donkey anti-goat antibody (Cat #A32814, Lot # UI289709, dilution 1:200, ThermoFisher Scientific)

	Validation statement of manufacturer: https://www.thermofisher.com/antibody/product/Goat-anti-Rabbit-IgG-H-L-Highly-Cross-Adsorbed-Secondary-Antibody-Polyclonal/A32814<br>-Alexa Fluor 546-conjugated donkey anti-rabbit antibody (Cat #A10040, Lot #948483, dilution 1:200, ThermoFisher Scientific)<br>Validation statement of manufacturer: https://www.thermofisher.com/antibody/product/Donkey-anti-Rabbit-IgG-H-L-Highly-Cross-Adsorbed-Secondary-Antibody-Polyclonal/A10040<br>-Alexa Fluor 647-conjugated donkey anti-mouse antibody (Cat #A32787, Lot #UI291059 dilution 1:200, ThermoFisher Scientific)<br>Validation statement of manufacturer: https://www.thermofisher.com/antibody/product/Donkey-anti-Mouse-IgG-H-L-Highly-Cross-Adsorbed-Secondary-Antibody-Polyclonal/A32787
Validation	Validation of antibodies is provided by manufacturer, please see the information above in antibody section.

Eukaryotic cell lines

Policy information about cell lines

Cell line source(s)	All cell lines are patient origin. GSLC NCH421k were generous gift of prof. Christel Herold-Mende (Heidelberg University, Heidelberg, Germany). GS025 GSLCs were isolated from patients with GBM at University of California Los Angeles (UCLA). Oral squamous carcinoma stem cells (UC2) were isolated from cancer patients with tongue tumor at UCLA and cultured as described previously. Primary immune cells were purified and isolated from peripheral blood of healthy donors and glioblastoma patients.
Authentication	Authentication of cells was performed by DNA fingerprinting using short tandem repeat (STR) analysis and amp-FlSTR Profiler Plus PCR Amplification Kit.
Mycoplasma contamination	All cells were tested negative for Mycoplasma using MycoAlert Mycoplasma Detection Kit (Lonza, Switzerland).
Commonly misidentified lines<br>(See ICLAC register)	No commonly misidentified cells were used.

Animals and other organisms

Policy information about studies involving animals; ARRIVE guidelines recommended for reporting animal research

Laboratory animals	Immune-deficient NSG mice (NOD scid gamma mouse, Jackson Laboratory, USA), female, 3 weeks old
Wild animals	NA
Field-collected samples	NA
Ethics oversight	University of California Los Angeles (UCLA). Animal research was performed under the written approval of the UCLA Animal Research Committee (ARC) (protocol # 2012-101-13).

Note that full information on the approval of the study protocol must also be provided in the manuscript.

Human research participants

Policy information about studies involving human research participants

Population characteristics	Only patients, diagnosed with astrocytoma grade IV, glioblastoma (GBM), are included in the study. Altogether, 8 patients with GBM were included. GBM biopsies were obtained from GBM patients who were operated at the Department of Neurosurgery, University Medical Center of Ljubljana, Slovenia. 7 of them are male and 1 is female. Their average age is 57 years. Details of GBM patients and their tumors are described in Supplementary Table 2. Healthy donors and patients with GBM for immune cell isolation from peripheral blood were recruited at UCLA. Samples of the donors of the same gender (male or female) were compared.
Recruitment	Glioblastoma biopsies were obtained from diagnosed glioblastoma patients who were operated at the Department of Neurosurgery, University Medical Center of Ljubljana, Slovenia. Details of GBM patients and their tumors are described in Supplementary Table 2. Healthy donors and patients with GBM for immune cell isolation from peripheral blood were recruited at UCLA.
Ethics oversight	The study was approved by the National Medical Ethics Committee of the Republic of Slovenia<br>(Approval no. 0120-190/2018/4).<br>The studies with immune cells from healthy donors and GBM patients were approved by the UCLA Institutional Review Board (IRB#11-000781), and all participants signed written informed consent in accordance with the Declaration of Helsinki.

Note that full information on the approval of the study protocol must also be provided in the manuscript.

Flow Cytometry

Plots

Confirm that:

☒ The axis labels state the marker and fluorochrome used (e.g. CD4-FITC).

☒ The axis scales are clearly visible. Include numbers along axes only for bottom left plot of group (a 'group' is an analysis of identical markers).

☒ All plots are contour plots with outliers or pseudocolor plots.

☒ A numerical value for number of cells or percentage (with statistics) is provided.

Methodology

Sample preparation	-Surface immunostaining of PBMCs, NK cells and GBM cells<br>PBMCs were isolated and NK cells were purified from healthy donors with no clinical signs of disease and patients diagnosed with glioblastoma using EasySep Human NK Enrichment kit (STEMCELL technologies, Canada). Glioblastoma stem-like cells (GSLCs) NCH421k were generous gift of prof. Christel Herold-Mende (Heidelberg University, Heidelberg, Germany) (references 3, 40). GS025 GSLCs were isolated from patients with GBM at University of California Los Angeles (UCLA) (reference 14). GS025 and NCH421k cells were grown in serum-free conditions as described before and were validated to express GSLC markers, such as CD133 and SOX2 (references 3, 14, 29, 41). Oral squamous carcinoma stem cells (UC2) were isolated from cancer patients with tongue tumor at UCLA and cultured as described previously (reference 23).<br>PBMCs, primary NK cells or GBM cells were harvested, washed with PBS and labelled with specific antibodies in 1 %BSA PBS and incubated for 30 min at 4�C. After washing with PBS samples were analyzed by Attune flow cytometer and FlowJo software.<br>-Intracellular staining<br>GBM cells were harvested, washed with PBS, fixed using fixation buffer (Biolegend) for 45 min at room temperature, and followed by permeabilization in permeabilization buffer (Biolegend) for 10 min at room temperature. Cells were labelled with specific primary antibodies in 1 %BSA PBS for 30 min at 4�C, washed with PBS, followed by secondary antibody incubation for 30 min at 4�C. After washing with PBS, samples were analyzed by Attune flow cytometer and FlowJo software.<br>-Cell death analysis<br>GBM cells were harvested, washed with PBS and propidium Iodide (PI) in final concentration of 10 �g/mL was added to cell suspension to evaluate the cell death. After 5 min incubation cell death was evaluated by Attune flow cytometer and FlowJo software.<br>-3D cell death analysis and surface marker analysis<br>GBM cells were labelled with 30 µM of CellTracker Green for 30 min. Then GBM cell tumorospheres were established as described previously (references 3 and 42) and incubated for 6 days in cell incubator. Primary and super-charged NK cells were labelled with 10 µM of CellTracker Blue for 30 min before they were added to GBM cell tumorospheres in different ratios in U-bottom 96-well plate for 4-48h. Tumorospheres were dissociated using mixture of TrypLE Express and Collagenase type II (5 mg/mL) in 2:1 ratio for 30 min and washed cells were stained to asses cell death using PI and flow cytometry and marker surface expression using immunolabelling and flow cytometry as described above. Number of dead cells was determined as % of CellTracker green-gated dead cells (PI-positive) and multiplied with number of GSLCs in control spheroids.
Instrument	Attune NxT Acoustic Focusing Cytometer flow cytometer (Invitrogen, Thermo Fisher Scientific, USA).
Software	FlowJo software v10 (Ashland, OR, USA) was used for data analyses.
Cell population abundance	Cells were not sorted. 10,000 cells per specific cell population, please see bellow
Gating strategy	-Surface immunostaining of PBMCs and NK cells<br>CD45(PE-Cy5)/SSC-H gating was performed to gate CD45-positive cells, followed by FSC-A/FSC-H gating. Cells were then gated in BL1/BL2 or BL3/BL2 channels to determine the percentage of positively labeled cells. Gates were set based on isotypic controls. 10,000 cells within CD45-positive cell population were analyzed to determine the percentage of immune cells in samples.<br>-intracellular and surface staining of GBM cells<br>FSC-H/SSC-H gating was performed to exclude cell debris, followed by FSC-A/FSC-H gating to analyze only singlets. Only singlets were then analyzed to evaluate mean fluorescence intensity of PE in BL2 channel. 10,000 cells within singlet cell population were analyzed for surface marker expression detection in GBM cells. Gates were set based on isotypic controls and mean fluorescence intensity of all samples was determined.<br>-Cell death analysis in cell culture<br>All cell events in FSC-H/SSC-H dot plot were analyzed for PI-positive cell population in channel BL2 (10,000 events). Gates were set on unstained cell population (PI-negative). In this control, less than 1 % of cells were PI-positive.<br>-3D cell death analysis and surface marker analysis<br>FSC-H/SSC-H gating was performed to exclude cell debris, followed by FSC-A/FSC-H gating to analyze only singlets. Only singlets were then gated based on CellTracker green (BL1 channel) and blue (VL1 channel) staining to determine GBM cells and NK cells, respectively. Gates to analyze GBM cells were set on unstained GBM cell population (CellTracker green- and CellTarcker blue-negative cells). In this control, less than 1 % of cells were CellTracker green- and CellTRacker blue-positive. Mean fluorescence intensity of surface markers was determined only in CellTracker-positive and CellTracker Blue-negative cell population (10,000 cells analyzed per sample). Gates for surface marker expression in GBM cells were set based on isotypic controls and mean fluorescence intensity of all samples was determined.<br>Percentage of PI-positive cells was determined in CellTRacker green-positive and CellTRacker blue-negative cell population

(10,000 cells analyzed per sample in BL2 channel). Gates for cell death analysis were set on unstained cell population (PI-negative). In this control, less than 1 % of cells were PI-positive.

☒ Tick this box to confirm that a figure exemplifying the gating strategy is provided in the Supplementary Information.
